# Advanced Echocardiography and Myocardial Work in Idiopathic Carpal Tunnel Syndrome with Presumptive Concurrent Cardiac Amyloidosis: An Exploratory Cross-Sectional Study

**DOI:** 10.3390/diagnostics16193124

**Published:** 2026-09-25

**Authors:** Raquel Machado, Sara Maia, Emanuel Oliveira, Pedro Palma, Helena Moreira, Laura Vieira, Manuela Fonseca, Carla Sousa, Ana Martins, Sofia Pimenta, Janete Santos, Elisabete Martins

**Affiliations:** 1 Faculdade de Medicina, Universidade do Porto, 4200-319 Porto, Portugal; 2 Escola Superior de Saúde, Universidade Técnica do Porto, 4200-072 Porto, Portugal; 3 Department of Cardiology, Centro Hospitalar Universitário do São João, 4200-319 Porto, Portugalelisabetemartins09@gmail.com (E.M.); 4 Department of Rheumatology, Centro Hospitalar Universitário do São João, 4200-319 Porto, Portugal; 5 RISE-Health, Faculdade de Medicina, Universidade do Porto, 4200-319 Porto, Portugal

**Keywords:** transthyretin cardiac amyloidosis, advanced echocardiography, myocardial work, speckle-tracking echocardiography, carpal tunnel syndrome

## Abstract

**Background/Objectives**: Idiopathic carpal tunnel syndrome (CTS) can frequently precede the clinical diagnosis of transthyretin cardiac amyloidosis (ATTR-CA). This study aimed to comprehensively characterize the standard and advanced echocardiographic phenotype in a screening cohort of patients with CTS assessed for concurrent ATTR-CA. **Methods**: Thirty-three patients with idiopathic CTS underwent comprehensive standard and advanced echocardiography. The presence of concurrent ATTR-CA was assessed using ^99m^Tc-DPD scintigraphy, allowing the stratification of the cohort into an amyloidosis group and a non-amyloidosis control group for comparative analysis. **Results**: Based on scintigraphy, 8 patients (24.2%) were diagnosed with presumptive ATTR-CA, acknowledging that AL amyloidosis was not systematically excluded, while 25 served as scintigraphy-negative controls. Standard echocardiography revealed greater wall thickness and worse diastolic function (*p* < 0.001) in the scintigraphy-positive group. Advanced imaging demonstrated profound left ventricular (LV) mechanical dysfunction, with severely reduced global longitudinal strain (*p* < 0.001) and a high prevalence of apical sparing (*p* < 0.001). Non-invasive myocardial work (MW) analysis showed a marked reduction in Global Constructive Work (*p* = 0.005) and Global Work Index (*p* = 0.017) in scintigraphy-positive patients, despite a relatively preserved LV ejection fraction. Multi-chamber strain analysis unmasked a pan-cardiac myopathy in the amyloid group, characterized by severe impairment of right ventricular (RV) global strain (*p* = 0.004) and profound depression of both left (*p* = 0.002) and right (*p* < 0.001) atrial reservoir strains. **Conclusions**: In patients with CTS, positive scintigraphy is characterized by severe, afterload-adjusted LV mechanical failure and profound biatrial and RV dysfunction. Advanced multi-chamber strain and MW indices show potential as non-invasive markers to help identify previously unrecognized pan-cardiac amyloid infiltration, guiding prompt diagnostic referral.

## 1. Introduction

Transthyretin cardiac amyloidosis (ATTR-CA) is an increasingly recognized cause of heart failure in older adults, resulting from progressive myocardial deposition of misfolded transthyretin (TTR) [1]. Despite growing awareness, the condition remains underdiagnosed, and early recognition is essential to maximize the benefit of disease-modifying therapies and improve clinical outcomes [1,2,3].

Carpal tunnel syndrome (CTS) has emerged as a key extracardiac manifestation of transthyretin amyloidosis, frequently preceding cardiac involvement by several years [4]. Bilateral idiopathic CTS in older individuals is increasingly recognized as a clinical red flag for ATTR-CA, reflecting early transthyretin deposition in tenosynovial tissue [5]. This temporal relationship, with musculoskeletal manifestations often preceding overt cardiac disease by five to nine years, creates a potential window for early detection, positioning patients with CTS as a high-risk population for targeted cardiac screening strategies [4].

Echocardiography is usually the first test performed in patients presenting with heart failure and suspected amyloidosis, defining the effects of amyloid burden through assessment of wall thickness, chamber dimensions, and diastolic dysfunction [6,7]. However, typical features are most prominent in advanced disease and may be missed in earlier stages [6]. Speckle-tracking echocardiography (STE) has enhanced diagnostic accuracy through the assessment of global longitudinal strain (GLS), which is typically reduced in ATTR-CA despite preserved ejection fraction [8], and the characteristic pattern of relative apical sparing (AS) has emerged as a non-invasive marker of cardiac amyloidosis [9]. However, AS has significant limitations as a screening tool, being present in approximately one-third of non-amyloid patients with similar clinical presentations, including those with severe aortic stenosis and end-stage renal disease [10,11]. Nevertheless, GLS remains load-dependent [12] and may not fully capture early myocardial dysfunction [8].

Myocardial work (MW) analysis represents a novel echocardiographic approach that integrates myocardial deformation with afterload through non-invasive pressure–strain loops, providing a more physiologically comprehensive assessment of ventricular performance than strain alone [13,14]. The four derived parameters, global work index (GWI), global constructive work (GCW), global wasted work (GWW) and global work efficiency (GWE), quantify myocardial energetics and mechanical efficiency [14], and are significantly more impaired in ATTR-CA compared with other hypertrophic phenocopies such as hypertrophic cardiomyopathy [15]. Emerging evidence further supports their ability to detect subtle contractile dysfunction and provide incremental diagnostic information beyond conventional strain analysis [12,15]. Despite this, the role of MW indices in the early detection of ATTR-CA, particularly in high-risk populations such as patients with idiopathic CTS, remains largely unexplored.

In parallel, cardiac amyloidosis is increasingly recognized as a multi-chamber disease, with diffuse involvement of the atria and right ventricle contributing to disease progression and prognosis [16]. Left atrial (LA) dysfunction in ATTR-CA is characterized by profound impairment of atrial strain, reflecting the progressive loss of atrial compliance due to amyloid infiltration, which has been shown to correlate with disease severity and worse clinical outcomes [17,18]. Similarly, right atrial (RA) strain is significantly reduced in cardiac amyloidosis, with reservoir and conduit strain significantly associated with mortality and showing potential utility for risk stratification [19]. Right ventricular (RV) involvement is equally relevant, with RV free wall longitudinal strain (RVFWLS) shown to be reduced in ATTR-CA, reflecting amyloid infiltration beyond the left heart and carrying independent prognostic significance [20]. Advanced echocardiographic assessment incorporating multi-chamber strain analysis, encompassing both atrial and RV deformation, may therefore substantially enhance early detection and risk stratification beyond left ventricular (LV) parameters alone, yet its application in a CTS screening context has not been systematically evaluated.

Bone tracer scintigraphy with technetium-labelled compounds has transformed the diagnostic pathway of ATTR-CA, enabling non-invasive confirmation of the disease with near-perfect positive predictive value in the absence of clonal dyscrasia [20]. However, identifying which patients should be referred for scintigraphy evaluation remains a key clinical challenge, particularly in populations presenting with extracardiac manifestations such as idiopathic CTS, where cardiac involvement may be subtle or entirely pre-symptomatic, making clinical recognition difficult [21]. Real-world data have shown that diagnostic delays remain substantial, with time from initial suspicion to confirmed diagnosis ranging from several months to over one year, underscoring the need for targeted screening strategies in high-risk populations [22].

Accordingly, this study aimed to investigate the potential association of advanced echocardiographic parameters, including GLS, MW indices and multi-chamber strain with the presence of ATTR-CA in patients with idiopathic CTS, hypothesizing that these parameters may improve the identification of previously unrecognised disease in this high-risk population.

## 2. Materials and Methods

### 2.1. Study Design and Population

This study was conducted as a sub-analysis of the CarPoS study (NCT05409833), a prospective, cross-sectional clinical study whose initial case series and overarching rationale have been previously described [23], aimed at investigating the association between CTS and ATTR-CA. Patients with symptomatic bilateral idiopathic CTS were recruited from the rheumatology consultation at São João University Hospital (Porto, Portugal) between July 2020 and July 2026.

For this specific advanced echocardiographic sub-study, a subset of 36 patients from the main cohort was selected to undergo comprehensive speckle-tracking and MW analysis between October 2025 and February 2026. At the time of this sub-study, the complete CarPoS cohort consisted of 54 eligible patients. Recruitment for the echocardiographic evaluation was non-consecutive. The remaining 18 eligible patients did not undergo advanced echocardiography primarily due to patient unavailability. Regarding the selection process, priority was given to patients who had not yet undergone baseline echocardiographic evaluation, followed by patients who had previously undergone a standard echocardiogram at the time of their initial study inclusion in previous years. Three patients were excluded due to poor acoustic windows precluding adequate echocardiographic evaluation, resulting in a final cohort of 33 patients.

Patients were excluded if they had secondary causes of CTS (such as chronic renal failure under hemodialysis, inflammatory arthropathies, gout, chondrocalcinosis, Colles’s fracture, space-occupying lesions, or infectious synovitis).

The study protocol was approved by the Ethics Committee for Health of São João University Hospital (No: CE 200/2020) and was conducted in accordance with the principles of the Declaration of Helsinki, the Convention on Human Rights and Biomedicine, and the guidelines of the Council for International Organizations of Medical Sciences. Written informed consent was obtained from all participating patients.

### 2.2. Clinical and Neurological Assessment

All patients underwent a thorough clinical evaluation, including medical history, physical examination, and an extensive laboratory work-up (assessing renal function, thyroid-stimulating hormone, rheumatoid factor, and antinuclear antibodies) to rule out secondary causes of CTS. The diagnosis of bilateral CTS was confirmed by electromyography/nerve conduction studies (categorizing involvement as mild, moderate, or severe) and wrist ultrasound. Wrist ultrasound was performed by the same experienced rheumatologist, who assessed the cross-sectional area of the median nerve at the tunnel inlet and evaluated for the presence of synovitis, tenosynovitis, and space-occupying lesions, ensuring the exclusive inclusion of idiopathic cases.

### 2.3. Cardiac Scintigraphy and Group Allocation

Because this study originated in a non-cardiac screening setting, the study protocol relied on Technetium-99m 3,3-diphosphono-1,2-propanodicarboxylic 76 acid (^99m^Tc-DPD) scintigraphy as the primary screening tool for ATTR-CA. Cardiac scintigraphy was performed 2 h after the intravenous administration of 20mCi of ^99m^Tc-DPD. The acquisition protocol included whole-body planar images, regional anterior, posterior, and lateral planar views of the thorax and wrists, and Single-Photon Emission Computed Tomography (SPECT) of the thoracic region. The integration of SPECT imaging was standard practice to accurately confirm myocardial localization and differentiate true myocardial uptake from intracavitary blood pool or adjacent rib uptake.

Scans were evaluated, and the Perugini grading (grades 0 to 3) was determined strictly on planar imaging by the same experienced nuclear medicine physician, who was completely blinded to the patients’ clinical data. Based on these findings, the cohort was stratified into two groups: the scintigraphy-positive group (defined by a Perugini grade ≥ 2) and the scintigraphy-negative control group (Perugini grade 0). Given the real-world screening nature of this study, comprehensive biochemical testing to exclude monoclonal gammopathy was not systematically performed in the entire cohort. Consequently, patients with significant myocardial uptake (Perugini ≥ 2) in the context of bilateral CTS were presumed to have ATTR-CA, acknowledging that the formal non-invasive exclusion of AL amyloidosis was not uniformly achieved.

### 2.4. Standard and Advanced Echocardiography

STE was performed offline using dedicated software (EchoPAC version 206, GE Vingmed Ultrasound AS). Images were acquired at optimized frame rates (40–80 Hz for the LV, 60–90 Hz for the RV, and 50–90 Hz for atrial strain). Both the primary analyst performing the advanced echocardiographic measurements and the supervising cardiologist validating the studies were completely blinded to the scintigraphy results. LV GLS was calculated from the standard apical views using the QRS onset as the reference point until automated aortic valve closure. The characteristic pattern of relative AS was systematically assessed qualitatively by visually identifying the classic “cherry-on-top” pattern on the longitudinal strain bull’s-eye plot. Advanced multi-chamber strain evaluation encompassed atrial strain across all three functional phases using the QRS onset (R-R gating) as the zero-reference point. Patients with inadequate acoustic quality or poor tracking in more than two segments in a single view were excluded from the analysis (yielding an overall feasibility rate of 91.7%, with 3 out of 36 initial patients excluded).

### 2.5. Myocardial Work Analysis

Non-invasive MW indices were derived from pressure–strain loops, which incorporate LV longitudinal strain data and an estimated non-invasive LV pressure curve. Brachial cuff blood pressure was measured three times, and the average value was used. Measurements were taken with the patient in the left lateral decubitus position immediately before the echocardiographic examination. The dedicated software provided four key parameters: GWI, representing total work performed during systole; GCW, representing work contributing to LV ejection; GWW, representing work not contributing to ejection; and GWE, calculated as constructive work divided by total work.

### 2.6. Statistical Analysis

Statistical analysis was performed using the software IBM SPSS Statistics (version 30.0). Given the small overall sample size and the unequal distribution between the study groups, a non-parametric statistical approach was adopted a priori for all comparisons. Continuous variables are expressed as median and interquartile range (IQR), and group comparisons (scintigraphy-positive vs. -negative) were performed using the non-parametric Mann–Whitney U test. Categorical variables are presented as absolute frequencies and percentages, with comparisons made using Fisher’s exact test. Effect sizes with 95% confidence intervals (CI) were calculated for all comparisons, utilizing Hodges–Lehmann estimates for median differences in continuous variables and Odds Ratios (OR) for categorical variables. Due to the small sample size and the exploratory nature of this study, no formal adjustments for multiple comparisons were applied. A two-sided *p*-value of <0.05 was considered statistically significant.

## 3. Results

### 3.1. Baseline Clinical and Conventional Echocardiography Characteristics

Of the initial CarPoS screening cohort, 33 patients with idiopathic CTS were included in the advanced echocardiographic analysis. Based on ^99m^Tc-DPD scintigraphy, 24.2% (*n* = 8) of the patients exhibited significant myocardial radiotracer uptake (Perugini grade ≥ 2) and were allocated to the scintigraphy-positive group. Importantly, because systematic biochemical evaluation for monoclonal gammopathy was not uniformly performed across the cohort, the possibility of AL amyloidosis in this subset cannot be definitively excluded. The remaining 25 patients, all of whom exhibited a Perugini grade of 0, constituted the scintigraphy-negative group.

Baseline clinical characteristics are summarized in Table 1. Patients in the scintigraphy-positive group were significantly older compared to the scintigraphy-negative group (median age 76 (7) vs. 64 (16) years, *p* = 0.036) and were predominantly male (88 vs. 32%, *p* = 0.012). Laboratory assessment revealed significantly higher Troponin I levels in the amyloidosis cohort (41.9 (60.5) vs. 2.6 (6.2) ng/L, *p* = 0.018).

Structurally, conventional echocardiographic assessment revealed a distinct infiltrative phenotype in the scintigraphy-positive group, characterized by significantly greater median IVS thickness (17.5 (6) vs. 12.0 (2) mm, *p* < 0.001) and increased indexed LA volumes (46.5 (13.8) vs. 27.8 (9.8) mL/m^2^, *p* < 0.001). Right heart structures were equally affected, with greater RV wall thickness (8.5 (3) vs. 4.0 (1) mm, *p* < 0.001). While LVEF remained preserved or only mildly reduced in both groups, it was slightly lower in the scintigraphy-positive patients (52.3 (19) vs. 56 (3) %, *p* = 0.003). Furthermore, diastolic dysfunction was more pronounced in the amyloid group, with a significantly elevated E/e’ ratio (11.5 (8.4) vs. 7.7 (3.3), *p* < 0.001).

### 3.2. Advanced Left Ventricular Deformation and Myocardial Work

The advanced echocardiographic and MW parameters are detailed in Table 2. Functionally, the scintigraphy-positive group was characterized by a profoundly impaired LV GLS (−7.9 (5.4) % vs. −16.2 (4.2) %, *p* < 0.001). The classic AS pattern was highly prevalent in the amyloidosis cohort, being present in 75% (*n* = 6) of scintigraphy-positive patients compared to only 4% (*n* = 1) in the scintigraphy-negative group (*p* < 0.001).

Non-invasive MW analysis provided further mechanical insights into LV performance. Patients with positive scintigraphy exhibited a significantly reduced GWI (708 (568) vs. 1527 (657) mmHg%, *p* = 0.017) and markedly lower GCW (897 (656) vs. 1965 (560) mmHg%, *p* = 0.005). There were no statistically significant differences observed between the two groups regarding GWW or GWE.

### 3.3. Multi-Chamber Strain Analysis

Beyond LV impairment, speckle-tracking analysis revealed severe biatrial and RV mechanical dysfunction in the scintigraphy-positive group (Table 2). Atrial reservoir function was markedly depressed in amyloid patients, evidenced by profoundly lower LASr (4.5 (17) vs. 24.0 (10) %, *p* = 0.002) and RASr (6 (18) vs. 26 (18) %, *p* < 0.001). Both conduit and contractile atrial phases were also significantly impaired. Additionally, RV mechanical dysfunction was prominent in the scintigraphy-positive group, with significantly impaired global RV longitudinal strain, including the septum (−10.8 (8.1) vs. −16.7 (5.6) %, *p* = 0.004).

## 4. Discussion

In this study, we comprehensively characterized the standard and advanced echocardiographic phenotype of patients with presumptive ATTR-CA within a screening cohort of individuals presenting with idiopathic CTS. The main findings of our study are: (1) advanced multi-chamber strain imaging unmasks profound subclinical mechanical dysfunction in scintigraphy-positive patients, extending far beyond the LV to include severe biatrial and RV impairment; (2) non-invasive MW indices, specifically GCW and GWI, are markedly depressed in amyloidosis; and (3) these advanced echocardiographic abnormalities are detectable and markedly abnormal in a targeted screening population, suggesting a potential value of advanced imaging in early disease recognition. It is crucial to emphasize that without the systematic exclusion of a plasma-cell dyscrasia in our cohort, the diagnosis remains presumptive, and the risk of misclassifying AL amyloidosis as ATTR-CA exists.

### 4.1. Left Ventricular Mechanics and Myocardial Work

Consistent with the previous literature, our presumptive ATTR-CA cohort exhibited the classic morphological features of cardiac amyloidosis, including increased wall thickness and overt diastolic dysfunction. However, the most striking differences were observed in functional myocardial deformation. LV GLS was severely reduced, and the characteristic AS pattern was present in 75% of the scintigraphy-positive patients. This pattern is a widely recognized hallmark of amyloid infiltration in current imaging guidelines [6,7,9], even though its diagnostic sensitivity can vary in multi-center real-world cohorts [11].

Beyond standard strain, our study adds valuable insights into the afterload-adjusted performance of the LV using non-invasive MW analysis. We observed a marked reduction in both GWI and GCW in the amyloid group. While GLS is inherently load-dependent, MW incorporates non-invasive blood pressure to better reflect actual energetic expenditure and contractility [13,14]. The substantial reduction in GCW (897 vs. 1965 mmHg%) highlights that the amyloid-infiltrated myocardium is intrinsically failing to generate effective mechanical work during systole, despite a relatively preserved LVEF. This aligns with recent data suggesting that MW indices, particularly GCW, are robust, afterload-adjusted markers of myocardial failure in ATTR-CA [12,15,24].

### 4.2. The Concept of Global Amyloid Myopathy: Atrial and Right Ventricular Involvement

Cardiac amyloidosis is a systemic disease, and our multi-chamber strain analysis heavily supports the concept of a “pan-cardiac” myopathy. The scintigraphy-positive patients demonstrated a marked decline in atrial function, particularly in the reservoir phase for both the left (LASr 4.5%) and right (RASr 6.0%) atria. Because atrial walls are thin, amyloid fibril deposition rapidly compromises their compliance and mechanical function, often before massive dilatation occurs. This severe biatrial mechanical failure has been shown to hold significant prognostic utility, explaining the disproportionately high thromboembolic risk classically associated with ATTR-CA, even in patients maintaining sinus rhythm [16,18]. Furthermore, RA impairment is increasingly recognized as a key marker of advanced disease [19]. Similarly, RV mechanics were significantly impaired. The reduction in global RV strain, alongside increased subcostal RV thickness, confirms that the right heart is not merely affected by backward failure consequent to LV diastolic dysfunction, but is also a direct target of amyloid infiltration, carrying significant prognostic weight [16]. The RVFWLS showed a similar magnitude of numerical difference between groups but did not achieve statistical significance (*p* = 0.067). Current consensus recommendations from the American Society of Echocardiography (ASE) and the EACVI advocate for RVFWLS as the primary parameter for assessing RV function, specifically to isolate true RV contractility from the mechanical tethering and infiltration of the shared interventricular septum [25]. In our cohort, the lack of statistical significance for RVFWLS, despite marked numerical impairment, likely reflects limited statistical power due to the small size of the scintigraphy-positive subgroup rather than a true absence of free wall involvement. Furthermore, given the extensive infiltration of the septum inherent to cardiac amyloidosis, the global RV strain parameter may capture this contiguous septal pathology more robustly in early or smaller cohorts, highlighting an important methodological nuance when applying current guidelines to infiltrative cardiomyopathies.

### 4.3. Clinical Implications in the CTS Cohort

CTS is a well-recognized early red flag for ATTR-CA, often preceding cardiac symptoms by several years [4,5]. Indeed, preliminary imaging data from our broader cohort have already highlighted a significant prevalence of previously unrecognized amyloid infiltration in this population [23]. In our current echocardiographic analysis, standard echo parameters were already abnormal in the presumptive ATTR-CA group. However, the use of advanced techniques like GLS, biatrial strain, and MW may serve as valuable early indicators. In patients presenting with orthopedic red flags, particularly those undergoing CTS release, the detection of profoundly reduced constructive work or atrial reservoir strain may raise clinical suspicion and support the consideration of further diagnostic evaluation, such as ^99m^Tc-DPD scintigraphy [21]. This proactive screening approach is vital to minimize the current real-world delays in ATTR-CA diagnosis [22], potentially opening the window for disease-modifying therapies, such as tafamidis, before irreversible heart failure ensues [2,26].

### 4.4. Limitations

This study has several limitations that must be acknowledged. Primarily, the overall sample size is relatively small, with 8 patients in the presumptive ATTR-CA group. This limited statistical power precluded the use of multivariate logistic regression models to adjust baseline differences. For instance, the scintigraphy-positive group was significantly older and predominantly male compared to the Non-CA group. Although this demographic profile is expected in wild-type ATTR-CA (1), age and sex are known independent confounders for myocardial strain, atrial mechanics and MW parameters. Consequently, our inability to statistically adjust for these baseline differences is a major limitation, and the unadjusted echocardiographic comparisons must be interpreted with caution, as the independent effect of amyloid infiltration cannot be entirely separated from demographic influences in this small cohort. Furthermore, because the advanced echocardiographic analysis was performed on a non-consecutive convenience subset of the overarching CarPoS cohort, there is an inherent selection bias. The logistical constraints preventing the inclusion of all eligible participants mean that this sub-study sample may not fully represent the broader screening population. Moreover, while effect sizes and CI have been reported, the remarkably wide intervals observed highlight the imprecision inherent to our small sample size. Therefore, the magnitude of these point estimates should be interpreted with caution, reinforcing the exploratory nature of this study.

Secondly, as this study was conducted within a real-world CTS screening cohort rather than a specialized amyloidosis clinic, comprehensive biochemical testing (including serum free light chains and serum/urine immunofixation) to exclude monoclonal gammopathy was not systematically performed in all patients. Current guidelines mandate the absence of monoclonal proteins to definitely diagnose ATTR-CA non-invasively using ^99m^Tc-DPD scintigraphy. Therefore, patients with Perugini grade ≥ 2 were presumptively classified as having ATTR-CA, and the lack of formal exclusion of AL amyloidosis in the entire cohort remains a recognized limitation of this study.

Genetic testing for TTR mutations was not systematically performed in all patients displaying myocardial uptake. Therefore, we were unable to definitively distinguish between wild-type and hereditary forms of ATTR-CA in our cohort. Nonetheless, the advanced age and marked male predominance of the presumptive ATTR-CA group strongly suggest a wild-type phenotype, which aligns with the classic epidemiological profile of patients presenting with bilateral idiopathic CTS.

Lastly, there are minor technical limitations regarding the advanced echocardiographic analysis. Relative apical sparing was assessed qualitatively via visual recognition of the classic longitudinal strain pattern rather than utilizing a formal quantitative ratio, which may influence its diagnostic sensitivity. Furthermore, formal intra- and interobserver reproducibility analyses for the advanced strain and myocardial work measurements were not systematically recorded in this specific sub-study cohort.

## 5. Conclusions

In conclusion, this study demonstrates that advanced echocardiography, encompassing multi-chamber strain and non-invasive MW, unveils profound and extensive, previously unrecognized, mechanical dysfunction in patients with presumptive ATTR-CA, identified through a CTS screening cohort. Given the methodological limitation of not systematically excluding AL amyloidosis, future studies with strict adherence to non-biopsy diagnostic guidelines are required to definitively confirm these findings. Despite a relatively preserved ejection fraction, the amyloid-infiltrated myocardium exhibits a dramatic decline in GCW, alongside severe biatrial and RV functional impairment. These findings reinforce the pathophysiological concept of ATTR-CA as a true pan-cardiac myopathy. From a clinical perspective, integrating these advanced echocardiographic parameters into the routine assessment of high-risk populations, such as those presenting with orthopedic red flags, may provide valuable exploratory indicators to aid in clinical decision-making. This approach may assist in raising clinical suspicion, potentially facilitating earlier diagnostic evaluation and ultimately widening the critical window for initiating disease-modifying therapies before the onset of irreversible heart failure.

## Figures and Tables

**Table 1:** Baseline clinical and conventional echocardiographic characteristics.

Variables	Scintigraphy-Positive (*n* = 8)	Scintigraphy-Negative (*n* = 25)	*p* Value	Effect Size [95% CI] *
Clinical and Laboratory Assessment				
Age (years), median (IQR)	76.0 (7)	64.0 (16)	0.036	−10 [−16, −1]
Male sex, *n* (%)	7 (88%)	8 (32%)	0.012	14.8 [1.6, 142.2]
BMI (kg/m^2^), median (IQR)	27.5 (6)	29.0 (7)	0.310	1 [−2, 5]
CTS symptom duration (years), median (IQR)	6.5 (9)	2.0 (4)	0.980	−4 [−8, 1]
Previous CV pathology, *n* (%)	5 (63%)	8 (32%)	0.213	3.5 [0.8, 18.6]
Heart failure, *n* (%)	3 (38%)	3 (12%)	0.137	4.4 [0.7, 28.6]
Atrial fibrillation, *n* (%)	2 (25%)	1 (4%)	0.194	6.4 [0.5, 86.3]
Hypertension, *n* (%)	8 (100%)	18 (72%)	0.154	-
Dyslipidemia, *n* (%)	6 (75%)	10 (40%)	0.118	4.5 [0.8, 26.9]
Troponin I (ng/L), median (IQR)	41.9 (60.5)	2.6 (6.2)	0.018	−32.5 [−69.1, −1.9]
BNP (ng/L), median (IQR)	216.1 (592.5)	54.0 (133.7)	0.073	−115.7 [−493.4, 16.8]
Creatinine (mg/dL), median (IQR)	0.88 (0.38)	0.77 (0.21)	0.352	−0.12 [−0.29, 0.10]
eGFR (mL/min/1.73 m^2^), median (IQR)	72.5 (30)	84.0 (16)	0.520	6 [−10, 22]
Blood Pressure				
SBP (mmHg), median (IQR)	126.5 (24)	126.0 (16)	>0.999	0.0 [−11, 17]
DBP (mmHg), median (IQR)	78.5 (19)	76.0 (8)	0.552	−2 [−8, 7]
LV and LA Morphology				
IVSd (mm), median (IQR)	17.5 (6)	12 (2)	<0.001	−6 [−8, −2]
PWd (mm), median (IQR)	15 (5)	12 (2)	<0.001	−4 [−6, −2]
RWT, median (IQR)	0.64 (0.35)	0.49 (0.16)	0.049	−0.11 [−0.29, −0.003]
iLV mass (g/m^2^), median (IQR)	80.5 (42)	66.5 (14)	0.029	−15 [−38, −3]
iLVtd vol (mL/m^2^), median (IQR)	69.9 (21.5)	57 (21.2)	0.046	−10.9 [−22.4, −0.9]
iLA bp vol (mL/m^2^), median (IQR)	46.5 (13.8)	27.8 (9.8)	<0.001	−17.9 [−26, −13.2]
RV and RA Morphology				
iRA vol (mL/m^2^), median (IQR)	32 (26.8)	19.6 (9.6)	0.004	−13.3 [−27.3, −4.4]
RV subcostal thickness (mm), median (IQR)	8.5 (3)	4 (1)	<0.001	−4 [−5, −3]
Hemodynamics and Function				
LVEF (%), median (IQR)	52.3 (19)	56 (3)	0.003	6.5 [1.3, 19.1]
CO (L/min), median (IQR)	4.5 (1.7)	3.6 (1.2)	0.404	−0.4 [−1.3, 0.4]
TAPSE (mm), median (IQR)	17 (1)	18 (3)	0.002	2 [1, 4]
S’ (cm/s), median (IQR)	10 (3)	13 (3)	0.041	2 [0, 4]
average e’ (cm/s), median (IQR)	6.3 (2.5)	7.8 (3.1)	0.007	1.8 [0.4, 3.6]
E/e’, median (IQR)	11.5 (8.4)	7.7 (3.3)	<0.001	−3.9 [−7.4, −2]
Valve disease, *n* (%)	1 (13%)	0 (0%)	-	-

* Effect sizes are reported as Hodges–Lehmann median differences for continuous variables (calculated as the scintigraphy-negative group minus the scintigraphy-positive group) and Odds Ratios for categorical variables (reflecting the odds in the scintigraphy-positive group relative to the scintigraphy-negative group). BMI, Body mass index; BNP, B-type natriuretic peptide; CO, Cardiac output; CV, Cardiovascular; CTS, Carpal Tunnel Syndrome; DBP, Diastolic blood pressure; eGFR, estimated glomerular filtration rate; iLA bp Vol, Indexed biplane left atrial volume; iLV mass, Indexed left ventricular mass; iLVtd vol, Indexed left ventricular end-diastolic volume; IQR, Interquartile range; iRA vol, Indexed right atrial volume; IVSd, Interventricular septal thickness at end-diastole; LA, Left atrium; LV, Left ventricular; LVEF, Left ventricular ejection fraction; PWd, Posterior wall thickness at end-diastole; RA, Right atrium; RV, Right ventricular; RWT, Relative wall thickness; SBP, Systolic blood pressure; TAPSE, Tricuspid annular plane systolic excursion.

**Table 2:** Advanced echocardiographic parameters: Strain and myocardial work.

Variables	Scintigraphy-Positive (*n* = 8)	Scintigraphy-Negative (*n* = 25)	*p* Value	Effect Size [95% CI] *
LV Deformation				
GLS LV (%), median (IQR)	−7.9 (5.4)	−16.2 (4.2)	<0.001	−7 [−9.4, −3.5]
AS, *n* (%)	6 (75%)	1 (4%)	<0.001	69 [5.3, 865.3]
Myocardial Work				
GWI (mmHg%), median (IQR)	708 (568)	1527 (657)	0.017	687 [223, 1009]
GCW (mmHg%), median (IQR)	897 (656)	1965 (560)	0.005	871 [471, 1225]
GWW (mmHg%), median (IQR)	91.5 (116)	118 (73)	0.578	12.5 [−43, 67]
GWE (%), median (IQR)	91.8 (8)	94 (6)	0.290	3 [−2, 6]
RV Strain				
RV global strain (%), median (IQR)	−10.8 (8.1)	−16.7 (5.6)	0.004	−5.7 [−9.6, −1.8]
RVFWLS (%), median (IQR)	−14.2 (10)	−19.3 (7)	0.067	−4.8 [−10.6, 0.5]
Atrial Strain				
LASr (%), median (IQR)	4.5 (17)	24 (10)	0.002	17.5 [4, 22]
LAScd (%), median (IQR)	−4.5 (4)	−10 (6)	0.002	−6 [−8, −2]
LASct (%), median (IQR)	0 (12)	−13 (10)	0.015	−10 [−15, −1]
RASr (%), median (IQR)	6 (18)	26 (18)	<0.001	17 [7, 25]
RAScd (%), median (IQR)	−5.5 (7)	−12 (11)	0.010	−7 [−13, −2]
RASct (%), median (IQR)	−0.5 (8)	−12 (9)	0.005	−9 [−13, −2]

* Effect sizes are reported as Hodges–Lehmann median differences for continuous variables (calculated as the scintigraphy-negative group minus the scintigraphy-positive group) and Odds Ratios for categorical variables (reflecting the odds in the scintigraphy-positive group relative to the scintigraphy-negative group). AS, Apical sparing; GCW, Global constructive work; GLS, Global longitudinal strain; GWE, Global work efficiency; GWI, Global work index; GWW, Global wasted work; LAScd, left atrial conduit strain; LASct, left atrial contractile strain; LASr, left atrial reservoir strain; LV, Left ventricular; RAScd, right atrial conduit strain; RASct, right atrial contractile strain; RASr, right atrial reservoir strain; RV, Right ventricular; RVFWLS, Right ventricular free wall longitudinal strain.

## Data Availability

The data presented in this article are not readily available because they are part of an ongoing study. Requests for access to these data should be made to the corresponding author, Raquel Machado, romachado@med.up.pt.

## Abbreviations

The following abbreviations are used in this manuscript:

^99m^Tc-DPD	Technetium-99m 3,3-diphosphono-1,2-propanodicarboxylic acid
AS	Apical sparing
ASE	American society of echocardiography
ATTR-CA	Transthyretin cardiac amyloidosis
CI	Confidence interval
CO	Cardiac output
CTS	Carpal tunnel syndrome
DBP	Diastolic blood pressure
EACVI	European association of cardiovascular imaging
eGFR	Estimated glomerular filtration rate
GCW	Global constructive work
GLS	Global longitudinal strain
GWE	Global work efficiency
GWI	Global work index
GWW	Global wasted work
IQR	Interquartile range
IVS	Interventricular septal
LA	Left atrial
LAScd	Left atrial conduit strain
LASct	Left atrial contractile strain
LASr	Left atrial reservoir strain
LV	Left ventricular
LVEF	Left ventricular ejection fraction
MW	Myocardial work
OR	Odds ratio
PW	Posterior wall
RA	Right atrial
RAScd	Right atrial conduit strain
RASct	Right atrial contractile strain
RASr	Right atrial reservoir strain
RV	Right ventricular
RVFWLS	Right ventricular free wall longitudinal strain
RWT	Relative wall thickness
SBP	Systolic blood pressure
SPECT	Single-photon emission computed tomography
STE	Speckle-tracking echocardiography
TAPSE	Tricuspid annular plane systolic excursion
TTR	Transthyretin
